# Optimization of Polydatin Hydrolysis Process Through Response Surface Methodology for Efficient Resveratrol Production

**DOI:** 10.3390/ph19050659

**Published:** 2026-04-23

**Authors:** Dong Wang, Yating Xiao, Xia Yang, Jie Cui, Yangyang Cai, Hua Chen

**Affiliations:** Key Laboratory of Protection, Development and Utilization of Medicinal Resources in Liupanshan Area, Ministry of Education, School of Pharmacy, Ningxia Medical University, Yinchuan 750004, China

**Keywords:** resveratrol, polydatin, acid-based hydrolysis, response surface methodology, high-performance liquid chromatography

## Abstract

**Background/Objectives:** Resveratrol is a naturally occurring polyphenolic stilbene compound exhibiting a wide range of biological activities, and it has been extensively utilized as both a food additive and a pharmaceutical active ingredient. Typically, it can be directly extracted from natural sources such as grapes, mulberries, and peanuts, or obtained through catalytic hydrolysis of polydatin. To establish an efficient and optimized method for resveratrol production, we conducted a comprehensive study to refine the acid-catalyzed hydrolysis conditions of polydatin. **Methods:** A high-performance liquid chromatography method was developed for the quantitative determination of polydatin and resveratrol. To identify the optimal ranges of reaction temperature, HCl concentration, and ethanol concentration, single-factor experiments were conducted by evaluating their influences on hydrolysis kinetics and resveratrol yield. Based on these results, response surface methodology incorporating a Box–Behnken design was employed to optimize the hydrolysis process, using resveratrol yield as the response variable. Furthermore, time-course experiments were performed to determine the optimal reaction duration under the established optimal conditions. **Results:** Single-factor experiments demonstrated that increasing temperature and HCl concentration significantly accelerated hydrolysis, but resveratrol yield increased initially and then decreased with excessive increases in either factor. To further optimize the process, response surface methodology optimization experiments were conducted at temperatures of 60, 70, and 80 °C; HCl concentrations of 1.0, 1.5, and 2.0 M; and ethanol concentrations of 75%, 85%, and 95%. The optimal conditions were identified as follows: temperature, 70 °C; HCl concentration, 1.5 M; ethanol volume fraction 85%; and reaction time, 5 h. Under these conditions, the theoretical resveratrol yield was 85.68%, and the average yield from triplicate validation experiments was 86.01% (RSD = 0.56%), which was consistent with the theoretical value. **Conclusions:** The optimized acid-catalytic hydrolysis process using RSM is stable, feasible, and efficient, offering a promising approach for enhancing resveratrol production from polydatin.

## 1. Introduction

Resveratrol, a plant-derived stilbene phytoalexin, whose biosynthesis and accumulation are typically induced in response to biotic or abiotic stress (e.g., pathogen attack, UV irradiation, or mechanical injury), has been researched extensively due to its diverse biological activities, including anti-aging, anti-tumor, anti-inflammatory, and cardioprotective effects [1,2,3,4]. Grapes, mulberries and peanuts are globally popular foods consumed both fresh and processed into products such as wine, juice, dried fruit, and jam. These foods are rich in essential nutrients (vitamins, trace elements, and dietary fiber) and bioactive compounds, particularly resveratrol [5]. With the increasing demand for natural health products, the market demand for resveratrol as a food additive and pharmaceutical active ingredient continues to grow annually.

In general, resveratrol can be directly extracted from natural sources such as grapes, mulberries, and peanuts, or obtained through chemical synthesis. Chemical synthesis entails multiple reaction steps and raises significant environmental concerns [6], whereas plant-based extraction is consistent with green production principles and facilitates the valorization of agricultural by-products (e.g., grape pomace, peanut skins) [7,8]. However, in plants, resveratrol predominantly occurs in the form of polydatin (resveratrol-3-O-β-D-glucoside). Polydatin acts as a prodrug that needs to be converted into resveratrol in the body to exert its pharmacological effects [9]. A previous study has shown that the bioavailability of polydatin in grape juice is significantly lower than that of resveratrol when orally administered to humans [10]. Moreover, the low natural abundance of free resveratrol limits extraction yields and increases production costs. As such, efficient conversion of polydatin to resveratrol is critical for improving resveratrol production efficiency.

The main methods for this conversion include enzymatic hydrolysis, microbial fermentation, and acid-catalyzed hydrolysis. Enzymatic hydrolysis is favored for its mild conditions and high specificity but is restricted by high enzyme costs, poor stability, and easy inactivation [11,12]. Microbial fermentation offers simple operation and high yields but faces challenges in strain screening and long production cycles [13,14]. By contrast, acid-catalyzed hydrolysis is widely used for glycoside conversion due to its simplicity, low cost, and scalability for industrial applications [15,16].

To increase the yield of resveratrol from polydatin, we optimized the acid-catalytic hydrolysis of polydatin. Single-factor experiments were used to evaluate the effects of temperature, HCl concentration, and ethanol volume fraction on resveratrol yield. Response surface methodology (RSM) was then applied to refine the process parameters, and the optimal reaction time was determined. The findings provide a scientific basis for efficient resveratrol production from polydatin.

## 2. Results

### 2.1. Validation of the Developed HPLC Method

#### 2.1.1. System Suitability

**Figure 1:**
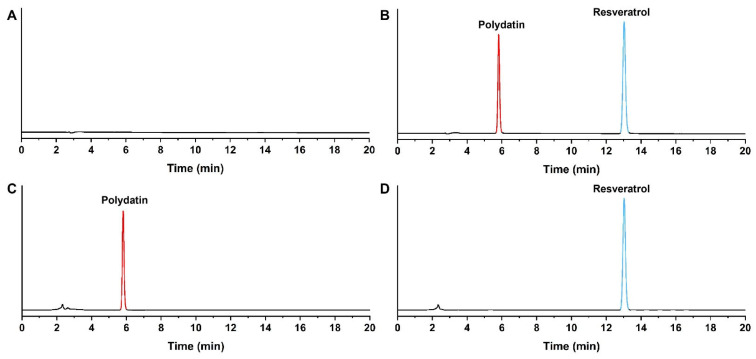
HPLC chromatograms of polydatin and resveratrol. (**A**) Blank solvent, (**B**) mixed reference solution of polydatin and resveratrol; (**C**) reaction solution before hydrolysis of polydatin; (**D**) test solution after complete hydrolysis of polydatin.

As shown in Figure 1, the theoretical plate number for resveratrol was more than 3000, the signal-to-noise ratio was more than 10, and the resolution was more than 1.5. No interference from other components was observed, confirming the method’s suitability for resveratrol quantification.

#### 2.1.2. Linearity

According to the calibration curve, a regression equation was derived as follows: Y = 1.14 × 10^5^X − 6970 (R^2^ = 0.9992). The results indicated a good linear relationship between resveratrol concentration (6.25–100 μg·mL^−1^) and peak area.

#### 2.1.3. Precision

The average concentration of resveratrol was 24.96 ± 0.42 μg·mL^−1^ (RSD = 1.69%), demonstrating excellent instrumental precision.

#### 2.1.4. Stability

The average resveratrol concentration over 24 h was 24.97 ± 0.65 μg·mL^−1^ (RSD = 2.61%), confirming the stability of the test solution under the storage conditions.

#### 2.1.5. Repeatability

The average concentration of resveratrol was 25.05 ± 0.65 μg·mL^−1^ (RSD = 2.61%), indicating good method repeatability.

#### 2.1.6. Recovery

The average recovery rate was 102.80% (RSD = 1.54%), confirming high method accuracy. The recovery rate was relatively high, exceeding the typically acceptable ±2% deviation range in pharmaceutical analysis. We believe the slightly elevated recovery rate may be attributed to the fact that a portion of HCl is consumed during the hydrolysis reaction, and heating may affect the ethanol concentration, ultimately leading to slight differences between the background of the reference solution and the test solution, which could result in a marginally higher recovery rate.

### 2.2. Optimization of Polydatin Hydrolysis

#### 2.2.1. Single-Factor Experiment

**Figure 2:**
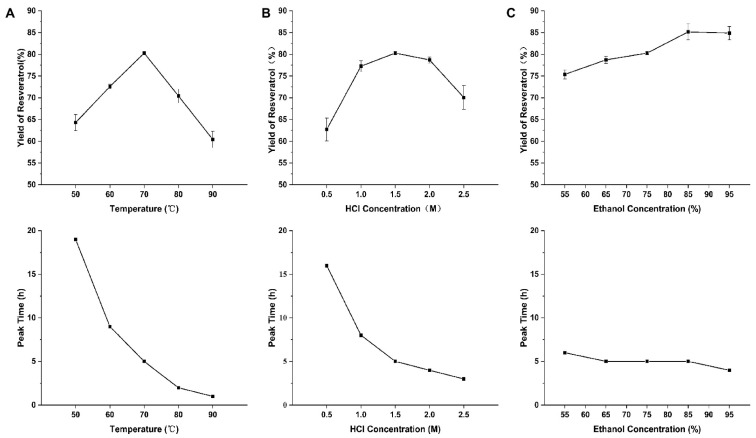
The effects of reaction temperature (**A**), HCl concentration (**B**), and ethanol concentration (**C**) on the yield of resveratrol and the time to peak yield.

Effect of temperature. Increasing temperature accelerated the hydrolysis reaction, reducing the time needed to reach maximum yield from 19 to 1 h. Resveratrol yield peaked at 70 °C (Figure 2A) and decreased at higher temperatures due to resveratrol degradation, confirming 70 °C as the optimal temperature.

Effect of HCl concentration. Increasing HCl concentration shortened the time to maximum yield from 16 h to 3 h. Yield peaked at 1.5 M and declined at higher concentrations, attributed to resveratrol instability under strong acid conditions, so 1.5 M was selected as the optimal HCl concentration.

Effect of ethanol concentration. Ethanol fraction moderately accelerated hydrolysis (time to peak yield reduced from 6 to 4 h). Yield increased with ethanol content and plateaued at 85%, possibly due to enhanced resveratrol stability in high-ethanol systems. Considering economy and efficiency, 85% ethanol was optimal.

#### 2.2.2. RSM Optimization

Factor selection. ANOVA of single-factor results showed that temperature, HCl concentration, and ethanol fraction all significantly affected resveratrol yield (*p* < 0.001), justifying their inclusion in RSM.

Regression modeling. Model fitting was performed based on the response surface experimental results (Table 1). The quadratic model was highly significant (*p* < 0.0001), with R^2^_Adj_ = 0.9875 and R^2^_Pre_ = 0.9433 (a difference less than 0.2), indicating excellent fit and predictability. The lack-of-fit test was non-significant (*p* = 0.3398 > 0.05), confirming model reliability. The regression equation for resveratrol yield was:

Y (%) = 85.68 + 5.61A + 5.69B + 0.995C − 8.64AB − 1.70AC − 1.42BC − 14.72A^2^ − 8.27B^2^ − 6.45C^2^

ANOVA results showed that factors A and B (main effects) were highly significant (*p* < 0.0001), while C was non-significant (*p* > 0.05) (Table 2), and interactions AB (*p* < 0.0001) and AC (*p* < 0.05) were significant, but BC was not (*p* > 0.05). Quadratic terms A^2^, B^2^, and C^2^ were all significant (*p* < 0.001), indicating nonlinear relationships. The order of factor importance was A ≈ B > C, and interaction significance was AB > AC > BC.

**Figure 3:**
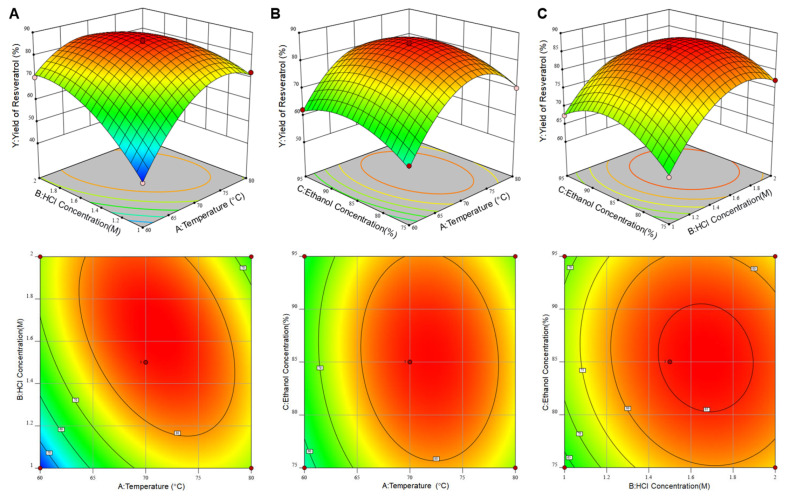
Response surface plots and contour plots illustrating the interaction among reaction temperature, HCl concentration and ethanol concentration, as well as their effects on the yield of resveratrol. (**A**) Interactions between reaction temperature and HCl concentration; (**B**) interactions between reaction temperature and ethanol concentration; (**C**) interactions between HCl concentration and ethanol concentration.

Response surface and contour plot analysis. Response surface plots (Figure 3) showed steep slopes for A and B, confirming their dominant role in yield regulation, while C had a milder effect (flat surface). Contour plots for AB and AC were elliptical, indicating strong interactions, whereas BC was nearly circular (weak interaction), consistent with ANOVA results.

Optimal parameters and validation. The predicted optimal parameters were 71.05 °C, 1.64 M HCl, and 85.32% ethanol, yielding a conversion rate of 86.80%. Adjusting for practical operation (70 °C, 1.5 M HCl, 85% ethanol), the theoretical yield was 85.68%. Validation experiments yielded an average yield of 86.01 ± 0.56%, which was within the 95% prediction interval, confirming model reliability.

**Figure 4:**
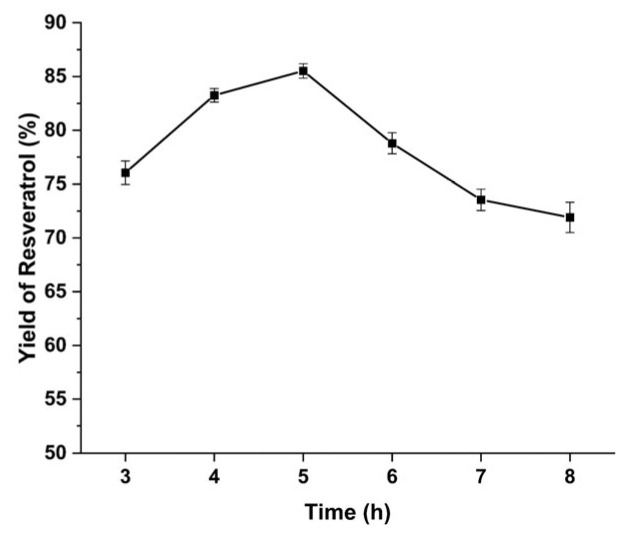
The influence of reaction time on the yield of resveratrol.

Optimization of reaction time. Under optimized conditions, resveratrol yield increased rapidly from 3 to 5 h, peaked at 5 h (86.01%), and declined thereafter (Figure 4), as resveratrol degradation exceeded formation. Thus, 5 h was determined as the optimal reaction time.

## 3. Discussion

Resveratrol is a natural product with diverse bioactivities, which drives the growing demand for resveratrol, but its low natural abundance limits production [7]. Some edible and medicinal plants, like *Polygonum cuspidatum*, contain free resveratrol and its glycoside polydatin. However, the content of free resveratrol in *Polygonum cuspidatum* is only 0.2–0.4%, whereas polydatin is present at approximately ten times the proportion of resveratrol [17]. Notably, in humans, intestinal epithelial cells can absorb only the aglycone form of resveratrol; thus, polydatin must first be hydrolyzed to resveratrol by β-glucosidase to enable efficient absorption. However, the bioavailability of glycosylated resveratrol (e.g., polydatin) in grape juice is substantially lower than that of the pure aglycone form [10,18,19]. As a direct active form, resveratrol remains irreplaceable in specific pharmacological pathways such as SIRT1 activation and anti-aging processes. Converting polydatin to resveratrol is therefore crucial for efficient utilization of natural resources.

Enzymatic, microbial and acid-based methods have been widely used in the hydrolysis of glycoside to aglycone [20]. Gong et al. achieved a 90.43% conversion of polydatin to resveratrol using β-glucosidase An3987 at 65 °C for 20 min [21], and Hu et al. reported a 93.1% conversion with *Bacillus subtilis* at 37 °C for 8 h [14]. Enzymatic hydrolysis offers notable advantages, including mild reaction conditions, high catalytic efficiency, and environmental compatibility. By contrast, microbial fermentation utilizes specific microbial strains to accomplish the biotransformation of glycosides, characterized by a straightforward process and high product yield. However, both enzyme and microbial preparations commonly face challenges such as high production costs, limited operational stability, and susceptibility to deactivation. Moreover, due to the low aqueous solubility of both resveratrol glycosides and their corresponding aglycone, reactions are typically carried out in solvent systems containing organic co-solvents. This imposes stringent demands on the organic solvent tolerance of enzymes and microorganisms, complicating strain and enzyme screening and resulting in prolonged development timelines, thereby hindering their industrial-scale application and commercialization. Under the optimized conditions in this study (70 °C, 1.5 M HCl, 85% ethanol, 5 h), the yield of acid-catalytic hydrolysis exceeded 86%. Although this is slightly lower than that of enzymatic and microbial methods, it offers the advantages of simple operation, low cost, and short development cycle, facilitating large-scale application. Acid-catalyzed hydrolysis is a viable alternative method. Li et al. used organic solvents such as acetone and methanol under acidic conditions to hydrolyze resveratrol glycosides, achieving a resveratrol yield of over 95% [15]. Liu et al. hydrolyzed *Polygonum cuspidatum* powder under the conditions of 65 °C, 8% HCl, 75% methanol, and a hydrolysis time of 5 h, obtaining a resveratrol yield of 2.06% [22]. However, previous studies have used toxic solvents such as methanol or acetone, which are not only environmentally unfriendly but also conflicts with green production standards.

Acid hydrolysis has been traditionally employed to convert polydatin to resveratrol. In the current study, ethanol was used as a green solvent, addressing environmental concerns while maintaining industrial applicability. We observed that after polydatin in the reaction solution was completely consumed, the actual yield of resveratrol was lower than the theoretical yield, suggesting the occurrence of side reactions or degradation of the generated resveratrol during the process. Therefore, it is necessary to strictly control the reaction conditions to identify the optimal process parameters. Single-factor experiments confirmed that increasing temperature and HCl concentration accelerate hydrolysis but induce resveratrol degradation at excessive levels. Ethanol enhanced yield by improving resveratrol stability, consistent with previous reports [23]. RSM optimization revealed significant interactions between temperature and HCl/ethanol, highlighting the need for multi-factor regulation. Wang et al. reported a method for preparing resveratrol from *Polygonum cuspidatum*, involving reflux extraction, filtration, acid- or enzyme-catalyzed hydrolysis to convert polydatin into resveratrol, liquid–liquid extraction, and chromatographic elution, yielding a final product with high recovery and a resveratrol content exceeding 73.8% [9]. However, this approach entails substantial equipment requirements and raises environmental concerns due to solvent consumption and energy intensity. In contrast, our optimized process operates under milder conditions and imposes significantly lower equipment demands.

The optimized process (70 °C, 1.5 M HCl, 85% ethanol, 5 h) offers advantages of simplicity, rapidity, and high yield (more than 86%), outperforming some existing acid hydrolysis methods. However, there were the following limitations: pure polydatin was used as the substrate, while plant extracts contain complex matrices that may interfere with hydrolysis. Future research will focus on direct hydrolysis of plant extracts to reduce purification steps and costs. Resveratrol instability during acid hydrolysis is another challenge. Studies have demonstrated that the stability of resveratrol is markedly affected by various environmental factors. Exposure to light, elevated temperatures, and oxidizing agents can all cause a substantial reduction in resveratrol content. Under conditions of light exposure or elevated temperature, resveratrol readily undergoes isomerization to its cis-isomer [24]; no corresponding chromatographic peaks were observed in this study. Moreover, previous research has indicated that resveratrol may undergo thermal oxidation, yielding dimers and various degradation products such as 3,5-dihydroxybenzaldehyde, 7-hydroxy-1-naphthaldehyde, 2-naphthalenemethanol, 3,4,3’,5’-tetrahydroxy-trans-stilbene, 2-methyltetrahydrofuran-3-one, and nonanoic acid [25]. Therefore, strict control of reaction temperature and duration is essential during the acid-catalyzed hydrolysis of polydatin to resveratrol, in order to prevent product degradation induced by overly harsh conditions. Novel technologies such as biphasic systems and deep eutectic solvents may mitigate this issue. Biphasic systems partition substrates and products, shifting equilibrium toward hydrolysis and reducing degradation [7], while deep eutectic solvent enhances the stability and yield of resveratrol [26,27]. Integrating acid hydrolysis with these technologies to develop one-pot extraction-conversion processes holds great promise for industrial application.

## 4. Materials and Methods

### 4.1. Materials and Reagents

Polydatin reference standard (purity > 99%) was purchased from Shanghai Aladdin Biotechnology Co., Ltd., Shanghai, China, and resveratrol reference standard (purity > 99%) was obtained from Shanghai Yien Chemical Technology Co., Ltd., Shanghai, China. Concentrated hydrochloric acid (37%, analytical grade), anhydrous ethanol (analytical grade), sodium hydroxide standard titration solution (1.0 M), methanol (HPLC grade), and acetonitrile (HPLC grade) were supplied by Chengdu Kelong Chemicals Co., Ltd., Sichuan, China, Shanghai Annegie Chemical Co., Ltd., Shanghai, China, Tanmo Quality Inspection Technology Co., Ltd., Beijing, China, and Thermo Fisher Scientific, Waltham, MA, USA, respectively.

### 4.2. Experimental Methods

#### 4.2.1. Quantification of Resveratrol

Chromatographic conditions. A Waters ARC high performance liquid chromatography (HPLC) system equipped with a 2998 photodiode array detector (Waters Corporation, Milford, MA, USA) was used for quantification. Separation was performed on a Shim-pack GIST-HP C_18_ column (4.6 mm × 250 mm, 5 μm; Shimadzu Research Laboratory Co. Ltd., Shanghai, China) at 35 °C. The mobile phase consisted of 0.1% formic acid in water (A) and acetonitrile (B) at a flow rate of 1.0 mL·min^−1^, and the gradient elution program was: 0–20 min, 77–67% A; 20–25 min, 67–77% A; 25–30 min, 77% A. The injection volume was 10 μL, and detection was performed at 306 nm. Each sample was measured in triplicate, and the results were averaged.

Preparation of standard solutions. Resveratrol reference standard (5.0 mg) was accurately weighed, dissolved in methanol, and diluted to 10 mL to prepare a 500 μg·mL^−1^ stock solution. Serial dilutions with methanol yielded calibration standards of 6.25, 12.5, 25, 50, and 100 μg·mL^−1^.

Preparation of test solutions. A 400 μL aliquot of the hydrolysis mixture was transferred to a pre-cooled 2 mL centrifuge tube and quenched in an ice-water bath; the low temperature markedly suppressed the hydrolysis rate, effectively halting the reaction. An equal volume of methanol was added to reduce the acidity while ensuring complete dissolution of resveratrol in the reaction mixture, followed by thorough vortex mixing and centrifugation at 8000 r·min^−1^ for 10 min. The supernatant was filtered through a 0.22 μm microporous membrane to obtain the test solution.

#### 4.2.2. Validation of HPLC Method

System suitability. Blank solvent, mixed standard solution (polydatin and resveratrol), pre-hydrolysis mixture, and post-hydrolysis test solution (10 μL each) were injected into the HPLC system. System suitability was evaluated based on theoretical plate number, signal-to-noise ratio, and resolution.

Linearity. Calibration standards were analyzed under the established chromatographic conditions. A calibration curve was plotted with peak area (Y) versus resveratrol concentration (X, μg·mL^−1^), and linear regression analysis was performed.

Precision. The 25 μg·mL^−1^ resveratrol standard solution was injected six times. Precision is expressed as the relative standard deviation (RSD) of the measured concentrations.

Stability. The test solution (under optimal hydrolysis conditions) was stored at room temperature in a sealed, light-protected container. Samples were analyzed at 0, 2, 4, 8, 12, and 24 h, and stability was evaluated by RSD of resveratrol concentrations.

Repeatability. Six replicate samples of polydatin (1.0 mg each) were prepared to 100 μg·mL^−1^, hydrolyzed under optimal conditions, and analyzed. Repeatability was determined by RSD of resveratrol yields.

Recovery. Six aliquots of test solution with known resveratrol concentration were spiked with an equal volume of resveratrol standard solution (similar concentration). The average recovery rate and RSD were calculated.

#### 4.2.3. Calculation of Resveratrol Yield

Resveratrol concentration in test solutions was determined by HPLC (triplicate analyses, average values). The yield (Y, %) was calculated using the following formula:

Y (%) = [(C_1_/M_1_)/(C_0_/M_0_)] × 100%

where C_1_ is the resveratrol concentration in the hydrolysis mixture (μg·mL^−1^), M_1_ is the molecular weight of resveratrol (228.24 g·mol^−1^), C_0_ is the initial polydatin concentration (μg·mL^−1^), and M_0_ is the molecular weight of polydatin (390.38 g·mol^−1^).

#### 4.2.4. Optimization of Hydrolysis Process

Polydatin hydrolysis procedure. Different ethanol solutions including 55%, 65%, 75%, 85% and 95% (*v*/*v*) were prepared with purified water. Concentrated HCl was diluted with the above ethanol solutions and calibrated using sodium hydroxide standard titration solution to adjust the HCl concentrations to 0.5, 1.0, 1.5, 2.0, or 2.5 M, respectively. Polydatin (1.0 mg) was dissolved in 10 mL of the acidified ethanol solution to obtain a reaction mixture, which was sealed and hydrolyzed in a thermostatic water bath at the selected temperature.

Single-factor experiments. The effects of temperature (50, 60, 70, 80 and 90 °C), HCl concentration (0.5, 1.0, 1.5, 2.0 and 2.5 M), and ethanol volume fraction (55%, 65%, 75%, 85% and 95%) on resveratrol yield were investigated (Table 3). Each factor was tested at five levels (triplicate per level), with the other two factors maintained at central levels. Samples were collected hourly to analyze hydrolysis kinetics and yield trends.

RSM analysis. Based on the single-factor experimental results, Design-Expert 12 software was employed to design a three-factor, three-level Box–Behnken experiment, with temperature (A), HCl concentration (B), and ethanol volume fraction (C) designated as input variables, and resveratrol yield (Y) as the response variable. The central levels were set at 70 °C, 1.5 M, and 85%, respectively, and the two levels adjacent to the central point were used as the low and high levels. A total of 15 experimental runs, including three replicates at the central point, each run was performed with three independent hydrolysis reactions. Regression modeling, analysis of variance (ANOVA), and response surface and contour plot analyses were performed to optimize the process parameters and evaluate the interactions among the factors. The optimized conditions were subsequently adjusted for practical feasibility and experimentally validated.

Determination of optimal reaction time. Samples were collected at 3, 4, 5, 6, 7, and 8 h under optimized conditions (70 °C, 1.5 M HCl, 85% ethanol). To determine the optimal reaction duration, three samples were prepared to assess resveratrol yield, with each sample analyzed in triplicate.

## 5. Conclusions

In this study, we optimized the acid-catalytic hydrolysis of polydatin to produce resveratrol using RSM. The optimal conditions were determined to be 70 °C, 1.5 M HCl, 85% ethanol, and a 5 h reaction time, yielding 86.01% resveratrol with high stability and reproducibility. The process uses ethanol as a green solvent, features simple operation and rapid kinetics, and offers significant potential for industrial-scale resveratrol production. Future work will focus on applying this process to plant extracts and integrating novel technologies to further improve yield and stability.

## Figures and Tables

**Table 1:** Box-Behnken response surface design and results.

Std	Factor A: Temperature (°C)	Factor B: HCl Concentration (M)	Factor C: Ethanol Concentration (%)	Response Y: Yield of Resveratrol (%)
1	60	1	85	42.36
2	80	1	85	72.44
3	60	2	85	70.21
4	80	2	85	65.74
5	60	1.5	75	57.16
6	80	1.5	75	70.21
7	60	1.5	95	62.21
8	80	1.5	95	68.45
9	70	1	75	62.28
10	70	2	75	77.29
11	70	1	95	67.45
12	70	2	95	76.79
13	70	1.5	85	86.09
14	70	1.5	85	86.43
15	70	1.5	85	84.52

**Table 2:** Variance analysis of quadratic model.

Source	Sum of Squares	Df	Mean Squares	F-Value	*p*-Value	Significance
Model	1912.53	9	212.50	123.73	<0.0001	***
A-Temperature	252.00	1	252.00	146.73	<0.0001	***
B-HCl Concentration	258.78	1	258.78	150.68	<0.0001	***
C-Ethanol Concentration	7.92	1	7.92	4.61	0.0845	NS
AB	298.43	1	298.43	173.76	<0.0001	***
AC	11.59	1	11.59	6.75	0.0484	*
BC	8.04	1	8.04	4.68	0.0828	NS
A^2^	799.91	1	799.91	465.76	<0.0001	***
B^2^	252.76	1	252.76	147.17	<0.0001	***
C^2^	153.79	1	153.79	89.55	0.0002	***
Residual	8.59	5	1.72	-	-	-
Lack of Fit	6.51	3	2.17	2.09	0.3398	NS
Pure Error	2.08	2	1.04	-	-	-
Cor Total	1921.11	14	-	-	-	-

* *p* < 0.05; *** *p* < 0.001; NS indicates *p* > 0.05.

**Table 3:** Experimental factors and levels.

Factor	Level 1	Level 2	Level 3	Level 4	Level 5
Temperature (℃)	50	60	70	80	90
HCl Concentration (M)	0.5	1.0	1.5	2.0	2.5
Ethanol Concentration (*v*/*v*)	55%	65%	75%	85%	95%

## Data Availability

The original contributions presented in this study are included in the article. Further inquiries can be directed to the corresponding author.
